# Catheter-Related Sepsis by *Candida pararugosa* in an Adult Patient under Chemotherapy Regimen

**DOI:** 10.1155/2021/8858157

**Published:** 2021-03-05

**Authors:** Gabriella Piatti, Sara Feltrin, Emanuela Fellini, Valentina Barbero, Alberto Ballestrero

**Affiliations:** ^1^Ospedale Policlinico San Martino, 10 Largo Benzi, Genoa 16132, Italy; ^2^Department of Surgical and Diagnostic Sciences (DISC), Section of Microbiology, University of Genoa, 8 Largo Benzi, Genoa 16132, Italy; ^3^Department of Internal Medicine and Medical Specialties (DiMI), University of Genoa, 8 Largo Benzi, Genoa 16132, Italy

## Abstract

*Candida pararugosa* is present in animals and humans in different organs and biological liquids, usually as a saprophyte. We report the case of a 61-year-old woman diagnosed with *de novo* stage IV metastatic lobular breast cancer, carrying a central venous catheter (port-a-cath) and bilateral stents for perirenal infiltration by malignancy. During chemotherapy regimen, a febrile episode occurred, along with a high level of serum glucan. The port-a-cath was removed after blood collection for culture, which gave isolation of *Candida pararugosa* strains. Given high glucan level and the patient's frailty, empirical treatment with fluconazole was started with load-dose, 800 mg orally, at day 1 and, afterwards, with 400 mg daily for two weeks. The phenotype of susceptibility to antibiotics of the strain demonstrated lower minimal inhibitory concentration to fluconazole than that reported in the literature. The patient remained asymptomatic, and inflammation parameters showed normalization. Unfortunately, three weeks later, meningeal localization of cancer caused rapid deterioration and death.

## 1. Introduction

*Candida pararugosa* are ascomycetes first isolated from human feces in 1978 [1]. Later, it was found in vegetable food [2–5] and anatomic sites of animals and humans [6, 7], where it is usually a harmless commensal organism and rarely involved in infections. A persistent detection of *C. pararugosa* from saliva of a two-year-old girl affected by rhabdomyosarcoma and relatives, and from the oral cavity of a healthy asymptomatic subject, was reported in 2004 [8, 9]. Recently, *C. pararugosa* was isolated from exterior infections, i.e., from onychomycosis [10]. The yeast was isolated as a cause of sepsis in Brazil and Qatar, respectively, in a child and in a newborn, in 2014 [11, 12]. Only one case report describes *C. pararugosa* isolated from blood and cause of sepsis in adult [13].

Since definition of *Candida* genus is highly polyphyletic and based on artificial morphologies, some species were moved to different genera, leading to a clinical linkage between phylogenetic placement and antifungal intrinsic resistance [7]. Interestingly, *C. pararugosa* belongs to fungi grouped as “rare yeasts” that, indeed, occur in low prevalence and express elevated minimal inhibitory concentrations (MICs) for at least one class of antifungals. These features make the microorganism worthy of particular attention, given the scarce clinical expertise and the difficulty of treatment [7].

## 2. Case Report

The patient was a woman in her sixties diagnosed with lobular breast cancer about six months earlier. She presented carcinomatous mastitis with lymph nodes infiltration of ipsilateral axillary region, widespread bone and liver metastasis, and perirenal infiltration by malignancy. Tru-Cut of breast lump demonstrated hormone-sensitive invasive lobular carcinoma. Immunohistochemistry assay showed ER = 90%, PR = 60%, Ki-67 = 75% Her2 neg (score 1+). The patient started first-line hormone-therapy with CDK 4/6 inhibitor (ribociclib) plus aromatase inhibitor (letrozole). In spite of two months therapy, the visceral disease progression and bilateral hydronephrosis necessitated the placement of bilateral stents and the beginning of first-line poly-chemotherapy with cyclophosphamide-epirubicin (EC75 q21) regimen and parental nutrition, after positioning of the central venous catheter (port-a-cath).

One week after the second of six courses of chemotherapy, the patient developed fever without apparent foci of infection. Laboratory blood tests showed normal leukocyte count (5.10/mm^3^) and low hemoglobin (82.0 g/L), while C-reactive protein (CRP) was 43.6 mg/L and erythrocyte sedimentation rate (ESR) was 109 mm/h. Central and peripheral blood cultures were repeatedly negative. Empirical antibiotic therapy with ceftriaxone and levofloxacin was undertaken, and the clinical response occurred after five days of treatment. In the suspicion of endocarditis, we performed echocardiography, which was negative for vegetation. Eleven days after the first febrile episode, the patient developed fever with normal leukocyte count (6.5/mm^3^), CRP of 20.5 mg/L, and serum glucan level of 128 pg/mL and the same of 183 pg/mL six days later. Consequently, bilateral stents, in spite of negativity of urine, were substituted and not cultured. Central and peripheral blood was inoculated in BD BACTECTM Plus Aerobic/F culture vials. Peripheral sample was also inoculated in Anaerobic/F vial and all were incubated in the automated system BD BACTED. Peripheral samples were negative. The Gram-stained slide obtained from positive central blood culture was allowed to observe yeasts presence. The respective vial was seeded on chromogenic agar (Chromatic™ Candida, Liofilmchem_srl_, Teramo-Italy) and blood and chocolate agar plates. After 24 hours, microbial growth was present in all plates as round, large, mucoid, pure colonies, and purple-colored in the chromogenic agar. The automated biochemical testing Vitek 2 (BioMeriéux Italia S.p.A., Grassina, Italy) failed the identification. The matrix-assisted laser desorption ionization time-of-flight mass spectrometry (MALDI-TOF) VITEK MS (BioMeriéux Italia S.p.A) gave *Candida pararugosa* identification. Given high glucan level and the patient frailty, the antifungal treatment was started immediately after collection for blood culture, with a load-dose of fluconazole of 800 mg at day 1 and, afterwards, with 400 mg daily for two weeks. The antifungal susceptibility testing was performed by the broth microdilution method using Sensititre™ YeastOne YO10 (TREK Diagnostic Systems, Ltd, United Kingdom) related to the MICs of the antifungal drugs shown in Table 1 and read visually as previously described [6]. The MIC of fluconazole for *C. pararugosa* isolate, repeated twice as the whole assay, was 2.0 mg/L.

Eye exam did not find fungal involvement of fundus oculi. The port-a-cath, removed after blood collection for culture and before the start of therapy, was grown and was negative, as well as the urine and the search for *C. pararugosa* in the stool, where we only found *Saccharomyces cerevisiae* as fungal microbiota. The patient recovered from fever on the first day of therapy with fluconazole and at the removal of the central catheter. She remained asymptomatic for infection, and serum glucan decreased during treatment until being negative at the end of therapy. CRP remained 20.0 mg/L probably for malignancy worsening. Two weeks after the fifth EC course, the patient developed a meningeal progression of cancer that leads to rapidly progressive deterioration of performance status and death.

## 3. Discussion

*Candida pararugosa* became a nosological entity in 2013, when the phylogenetic analysis excluded it from *C. rugosa* complex [14]. *C. pararugosa* remains present in the so-called group of “rare ascomycetous yeasts,” definition relevant from the clinical point of view [7]. Many identifications of *C. pararugosa* strains were likely lost when recognized as *C. rugosa* or *C. rugosa* species complex and when the MALDI-TOF system and sequencing methods were not available. Apart from the detection of *C. pararugosa* in many types of food and sporadic findings in humans, at least seventy-five *C. pararugosa* clinical isolates (between 2% and 20% of the total *Candida* strains) emerge from the antifungal resistance surveys of *Candida* species, which did not report the corresponding clinical features [6, 7, 15]. The yeast appears to be a nonpathogenic saprophytic yeast, causing only urinary infections and sepsis among immunocompromised patients. Only one clinical report describes the occurrence of sepsis due to *C. pararugosa* in adults by correlating the infection with the fungal detection, in 2017 [13].

In our report, the patient presented stage IV metastatic lobular breast cancer. She was under chemotherapy treatment, was carrying a port-a-cath central venous catheter and bilateral urinary stents, was fed with parental nutrition, and had been recently exposed to broad-spectrum antibacterial therapy. All these are clinical aspects worthy of being considered as predisposing factors for candidemia [6]. The lack of leukopenia makes it difficult to correlate the infection with chemotherapy, which can affect the bowel mucosa and favor invasion by the yeasts. Bilateral stents were replaced after detection of elevated serum glucan levels without being cultured. The urine was negative as was the search for *C. pararugosa* in patient's feces. Although the infection itself lasted a very short period and isolation of *C. pararugosa* occurred only from the central venous catheter, the latter was confirmed in the clinical relevance by high glucan levels. The port-a-cath had been placed sixty-six days prior to the second febrile episode, and its removal coincided with the start of therapy and resolution of fever. These circumstances made us think of a single day of fluconazole treatment as a chance event in relation to clinical resolution. Like other *Candida* species, *C. pararugosa* produces biofilms, especially in prosthetic materials [16]. Therefore, we think that the removal of the central catheter, along with possible biofilm, was instrumental in resolution of the infection, maintained with continuance of antifungal therapy. From the microbiological point of view, our report confirms the inability of biochemical testing to identify *pararugosa* species and the need to utilize molecular methods or spectrometry. Instead, we repeatedly obtained MIC values for both azole and echinocandin different from those described in the literature. In particular, MIC for fluconazole was lower than that obtained by Pérez-Hansen et al. and similar to that obtained by Paredes et al. [7, 15] and was supported by the persistence of the response to treatment.

## Figures and Tables

**Table 1:** Antifungal sensitivity phenotype of *Candida pararugosa* strain from the patient compared with pattern described in epidemiological surveys of literature.

Antifungal drug	Value mg/L^a^	Value mg/L^b^		Value *µ*g/mlc		
	MIC	MIC_50_	MIC_90_	Range	GM	MIC_90_
ITC	1.0	0.5	0.5	0.03–0.25	0.07	0.12
POS	0.12	0.25	0.5	0.03–0.12	0.08	0.12
ISA	—	0.5	1.0	—	—	—
FLC	2.0	16.0	32.0	0.25–2	0.76	2
VRC	0.12	0.5	1.0	<0.03–0.12	0.03	0.06
ANI	0.06	1.0	>4.0	0.03–0.12	0.06	0.12
MICA	0.12	0.25	>4.0	0.06–0.12	0.1	0.12
CAS	0.25	1.0	>4.0	0.03–0.25	0.13	0.25
AMB	1.0	0.5	1.0	0.25–1	0.47	1

ITC, itraconazole; POS, posaconazole; ISA, isavuconazole; FLC, fluconazole; VRC, voriconazole; ANI, anidulafungin; MICA, micafungin; CAS, caspofungin; AMB, amphotericin B. a, values obtained on *C. pararugosa* strain of this report; b, values obtained on isolates of reference [7]; c, values obtained on isolates of reference [6]. MIC_50_, the minimal concentration of drug capable of inhibiting the growth of 50% of assayed isolates. MIC_90_, the minimal concentration of drug capable of inhibiting the growth of 90% of assayed isolates. GM, geometric mean. —, value not calculated.
